# The association between severity of King’s Obesity Staging Criteria scores and treatment choice in patients with morbid obesity: a retrospective cohort study

**DOI:** 10.1186/s40608-016-0133-1

**Published:** 2016-12-07

**Authors:** Tone G. Valderhaug, Erlend T. Aasheim, Rune Sandbu, Gunn S. Jakobsen, Milada C. Småstuen, Jens K. Hertel, Jøran Hjelmesæth

**Affiliations:** 1 Morbid Obesity Centre, Vestfold Hospital Trust, Tønsberg, Norway; 2 Department of Endocrinology, Akershus University Hospital, Nordbyhagen, Norway; 3 Division of Medicine and Laboratory Sciences, Institute of Clinical Medicine, University of Oslo, Oslo, Norway; 4 Department of Health Management and Health Economics, University of Oslo, Oslo, Norway; 5 Imperial Weight Centre, Imperial College London, London, UK; 6 Department of Surgery, Vestfold Hospital Trust, Tønsberg, Norway; 7 Institute of Clinical Medicine, Faculty of Medicine, University of Oslo, Oslo, Norway; 8 Department of Endocrinology, Morbid Obesity and Preventive Medicine, Institute of Clinical Medicine, University of Oslo, Oslo, Norway; 9 Tone Gretland Valderhaug, Division of Medicine, Department of Endocrinology, Akershus University Hospital HF, Sykehusveien 25, 1478 Nordbyhagen, Norway

**Keywords:** Morbid obesity, Metabolic syndrome, Bariatric surgery

## Abstract

**Background:**

The King’s Obesity Staging Criteria (KOSC) comprises of a four-graded set of health related domains. We aimed to examine whether, according to KOSC, patients undergoing bariatric surgery differed from those opting for conservative treatment.

**Methods:**

We graded 2142 consecutive patients with morbid obesity attending our centre from 2005-10 into the following KOSC domains: airway/apnoea, body mass index (BMI), cardiovascular risk (CV-risk), diabetes mellitus, economic complications, functional limitations, gonadal dysfunction, and perceived health status/body image. Both patients and physicians agreed upon treatment choice through a shared decision making process.

**Results:**

A total of 1329 (62%) patients opted for lifestyle intervention and 813 (37%) for bariatric surgery as their first treatment choice. The patients treated with bariatric surgery were younger (42 vs. 44 years, *p* < 0.001), had a higher BMI (45.4 vs. 43.8 kg/m^2^, *p* < 0.001) and had a lower ten year estimated CV-risk (9.4 vs. 10.7%, *p* = 0.004) than the lifestyle intervention group. Compared with having BMI < 40 kg/m^2^, BMI ≥ 40 kg/m^2^ was associated with 85% increased odds of bariatric surgery (OR 1.85 [95% CI 1.48, 2.30]). Conversely, patients with ≥20% ten year CV-risk, had lower odds of bariatric surgery than patients with <20% CV-risk (0.68 [0.53, 0.87]).

**Conclusion:**

BMI was the strongest KOSC-domain associated with subsequent bariatric surgery after a shared decision making process. Prospective studies are required to assess whether the use of KOSC can help guide patients and clinicians to identify the most appropriate choice of treatment for morbid obesity.

## Background

Ways of measuring and describing health status in individual patients with obesity are required for several reasons. The increasing prevalence of obesity suggests that in order to optimize health gains at a societal level it is necessary to prioritize for treatment those patients who may benefit the most. At the level of the individual patient, patients and clinicians discussing treatment options will benefit from being able to consult robust evidence on the expected benefits and side-effects of different weight-loss treatments in patients with a given health profile.

The most widely used measure of obesity is the body mass index (BMI). Increasing BMI is associated with increased risks of type 2 diabetes, cardiovascular disease and incidence of several cancers [1–3]. However, BMI and other anthropometric classification systems neither accurately reflect the presence of nor the severity of obesity-related health risks, comorbidities or quality of life at the individual level [4]. Accordingly, two clinical staging systems for obesity-related conditions and comorbidities were recently proposed. The aims of these systems were to take a more holistic approach to describing health status in individual patients, and to help define a clear indication for obesity treatment as well as to identify patients who might benefit the most from bariatric surgery [5–8]. The Edmonton’s Obesity Staging System (EOSS) classifies patients with BMI ≥30 kg/m^2^ according to a medical, mental and functional axis [7]. The King’s Obesity Staging Criteria (KOSC) classifies patients into nine domains, within which each patient is assigned a stage from 0 to 3 [6, 9].

In this study, we aimed to retrospectively assess if, according to the KOSC, patients who underwent bariatric surgery at a tertiary care centre differed from those opting for non-surgical treatment. We hypothesized that patients who underwent bariatric surgery would have higher scores according to KOSC in most domains, indicating more obesity related co-morbidities.

## Methods

### Design and study population

Treatment seeking patients with morbid obesity who were referred from local hospitals to a tertiary care centre (the Morbid Obesity Centre, Vestfold Hospital Trust) in southern Norway and who accepted to be enrolled in the Registry- and Biobank study from November 28th 2005 until August 6th 2010, were assessed for eligibility. Of 2184 patients, 42 patients with BMI <35 kg/m^2^ did not fulfil the criteria for bariatric surgery and were excluded from the study, leaving 2142 treatment seeking patients eligible for bariatric surgery to be included in the final analysis. A total of 2075 (98%) of the study participants were Caucasians. Data was missing for the following KOSC domains: domain A; *n* = 10, domain C; *n* = 13, domain D; *n* = 3, domain E; *n* = 420, domain F; *n* = 582. The data representing domain E and domain F were included in the database from May 2007. The patients were provided comprehensive information about both the risks and benefits of different treatment methods. Together patients and physicians agreed upon the most appropriate choice of therapy; either bariatric surgery or intensive lifestyle intervention (shared decision making) [10, 11]. A total of 157 (7%) study participants treated with lifestyle intervention first and bariatric surgery thereafter were included in the lifestyle intervention group. The overall median (range) time from the first consultation until surgery was 21 (380) months. The wait time for surgery in the subgroup of patients who chose lifestyle intervention first and then bariatric surgery was longer than for patients who chose bariatric surgery first (median 37 [11–80] months vs. 19 [3–78] months, *p* < 0.001). The final patient included in the study underwent bariatric surgery on May 7th 2013. Treatment of morbid obesity is financed through the universal health care system in Norway. Thus, the patients were able to choose between intensive lifestyle intervention and bariatric surgery independent of their financial status. The study was approved by the Regional Committee for Medical and Health Research Ethics (S-05175). The participants provided written informed consent, and the study was performed in accordance with the Declaration of Helsinki [12].

### Data collection and definitions

Data on patient history of obesity related comorbidities were retrieved in a clinical setting, as described previously [13]. All anthropometric and blood pressure measurements were performed by trained study personnel. Blood pressure was measured with an appropriate cuff after at least 5 min rest with the patient seated in an upright position. Three measurements were registered and the average of the second and the third measurement was used in the study. We defined metabolic syndrome ﻿(MetS) according to the joint interim statement, of the International Diabetes Federation Task, Force on Epidemiology and Prevention; National Heart, Lung, and Blood Institute; American Heart Association; World Heart Federation; International Atherosclerosis Society; and International Association for the Study of Obesity (2009) [14]. The criteria for MetS were fulfilled if WC ≥ 80 cm (women) or ≥ 94 cm (men) combined with a minimum of two out of four criteria present: 1) low HDL-cholesterol; HDL-cholesterol <1.3 mmol/L (women)/ HDL-cholesterol <1.0 mmol/L (men), 2) hypertriglyceridemia; triglycerides ≥1.7 mmol/L, 3) raised blood pressure; systolic blood pressure ≥130 mmHg or diastolic blood pressure ≥85 mmHg, or use of blood pressure lowering medication, and 4) dysglycemia; fasting serum glucose ≥5.6 mmol/L or known diabetes mellitus. In addition, patients who used lipid lowering drugs fulfilled the lipid criteria for MetS, patients who used antihypertensive drugs fulfilled the blood pressure criteria for MetS and patients who used glucose lowering drugs fulfilled the glucose criteria for MetS.

### Applied kings obesity staging criteria

The applied KOSC are presented in Table 1, including the domains A; airway/apnoea, B; BMI, C; cardiovascular (CV) risk, D; diabetes mellitus, E; economic complications, F; functional limitation, G; gonadal dysfunction and HI; perceived health status, body image and eating behavior. Patients in the two lower stages (0-1) were assumed to have a minor risk of future morbidity and mortality. Furthermore, the higher stages (2-3) correlate with several clinical, metabolic and psychological conditions contributing to patient morbidity and mortality [9]. Domain C was defined by calculating the ten year CV-risk according to the Framingham risk score equation [14]. Domain E was defined according to working status as given by the public social benefit welfare system in Norway. Patients who were unable to work full time or were unemployed were classified as stage 2, and patients who received disability pension were classified as stage 3. We used levels of physical activity to define functional limitations (domain F), with less than one hour moderate or vigorous physically activity each week classified as stage 2 (physically inactive). Data on gonadal function were available for women only (domain G). Data on perceived health status and body image were combined to obtain the best possible data set (domain HI). Patients who reported anxiety or depression as baseline, but who did not use medication, were classified as stage 1. Those patients taking antidepressive or antipsychotic medication or had an eating disorder were classified as stage 2.

**Table 1:** King’s obesity staging criteria as applied in the 2142 consecutive treatment seeking patients with morbid obesity

Criteria		Stage 0 Normal health	Stage 1 At risk of disease	Stage 2 Established disease	Stage 3 Advanced disease
A	Airways	Normal Neck < 43 cm	Mild OSA Neck ≥ 43 cm Asthma/COPD	Requires CPAP	-
B	BMI	NA^a^	35-39.9 kg/m^2^	40-50 kg/m^2^	>50 kg/m^2^
C	CV-risk	<10%	10-19%	≥20% Stable CAD	
D	Diabetes	FPG < 5,6 HbA1 < 5,7	IFG HbA1c 5.7-6.4%	DM2 HbA1c < 9%	DM2 HbA1c ≥ 9%
E	Economic complications	None	None	Workplace disadvantage	Disabled
F	Functional limitation	≥3 h moderate physical activity/week	1-2 h moderate physical activity/week	<1 h moderate physical activity/week	-
G	Gonadal dysfunction^b^	Normal	Hyperandrogenemia^c^	PCOS^d^	-
H	Perceived Health status, body Image	Normal	Anxiety/depression without medication	Psycoactive drugs Eating disorder	-
I					

*OSA*, obstructive sleep apnea, *COPD*, chronic obstructive pulmonary disease, *CPAP*, continuous positive airway pressure, *BMI*, body mass index, CV-risk, ten years risk of cardiovascular disease (Framingham risk assessment), *CAD*, coronary artery disease, *FPG*, fasting plasma glucose, *IFG*, impaired fasting glucose, *DM2*, diabetes mellitus type 2, *PCOS*, polycystic ovarian syndrome

^a^ Patients with BMI < 35 kg/m^2^ (*n* = 42) were excluded from the analysis since they did not fulfil the criteria for bariatric surgery

^b^Female participants

^c^Hyperandrogenemia denotes a free testosterone index (FTI) above the normal range (FTI > 0.6). FTI was calculated by the formula 100 x serum testosterone (nmol/L) / sex hormone binding globulin (SHBG, nmol/L)

^d^Women with known PCOS and those with an FTI > 0.6 or hirsutism combined with oligo- / anovulation were classified as having PCOS

### Statistical analysis

Data are presented as mean (standard deviation [SD]) or proportions (%). Continuous variables were compared using independent samples *t*-test and categorical variable using either *χ* ^2^ test or Fisher’s exact test, as appropriate. Univariable and multivariable logistic regression analyses were used to assess the associations between KOSC and obesity treatment. In the risk assessment, we compared patients with lower risk of disease (stage 0-1) with patients with higher risk (stage 2-3). We also performed sub-analyses for domain B comparing stage 2 (BMI 40–50 kg/m^2^) and stage 3 (BMI > 50 kg/m^2^) with stage 1, reference (BMI 35–39,9 kg/m^2^) and for domain D comparing patients in stage 1 (IFG or HbA1c 5.7–6.4%), stage 2 (type 2 diabetes and HbA1c <9%) and stage 3 (type 2 diabetes and HbA1c ≥9%) with stage 0 (normal glucose metabolism, reference). *P*-values <0.05 were considered statistically significant. All analyses were performed using IBM SPSS statistics 22.

## Results

A total of 2142 consecutive treatment seeking patients were included in the analyses. Of these, 1329 (62%) patients chose lifestyle intervention and 813 (38%) opted for bariatric surgery (Table 2). Compared to those who underwent lifestyle intervention, the patients treated with bariatric surgery were approximately two years younger, had a higher BMI, had a higher proportion of current smokers as well as a lower ten year estimated risk of incident cardiovascular disease (CVD). The proportions of patients with obstructive sleep apnea (OSA), type 2 diabetes, hypertension, coronary artery disease and MetS did not differ significantly between groups (Table 2). Patients who developed obesity before 20 years of age were more likely to choose bariatric surgery first than the patients who developed obesity as adults (i.e. ≥20 years of age) (65% vs. 55%, *p* < 0.001).

**Table 2:** Characteristics of 2142 consecutive treatment seeking patients stratified by treatment choice

	Lifestyle	Bariatric surgery	*P* value
N	1329 (62%)	813 (38%)	-
Age, yrs	44 (13)	42 (11)	<0.001
Female gender	870 (66%)	548 (67%)	0.371
OSA	250 (19%)	151 (19%)	0.909
CPAP	183 (14%)	92 (11%)	0.110
COPD or Asthma	61 (5%)	29 (4%)	0.269
Waist circumference, cm	130 (14)	133 (14)	<0.001
BMI, kg/m^2^	43.8 (5.8)	45.4 (6.0)	<0.001
Hypertension	802 (60%)	498 (61%)	0.648
Ten year cardiovascular risk, %	10.7 (10.6)	9.4 (9.2)	0.004
Coronary artery disease	66 (5%)	30 (4%)	0.196
Current smoker	333 (25%)	239 (29%)	0.031
Metabolic syndrome	921 (69%)	588 (72%)	0.143
HbA1c, %	6.1 (2.2)	6.1 (1.3)	0.769
Type 2 diabetes	405 (31%)	253(31%)	0.772
Family history diabetes	429 (32%)	283 (35%)	0.237
Workplace disadvantages	274 (21%)	187 (23%)	0.194
Disabled	307 (23%)	162 (20%)	0.085
<1 h moderate physical activity / week	386 (39%)	198 (35%)	0.142
Hyperandrogenemia^a^	377 (43%)	230 (42%)	0.620
PCOS^a^	121 (14%)	72 (13%)	0.692
Anxiety/depression	542 (41%)	327 (40%)	0.821
Eating disorder	87 (7%)	54 (7%)	0.929
Psychoactive drug	243 (18%)	149 (18%)	0.954

Data are presented as mean (SD) or n (%). *OSA* Obstructive sleep apnea, *CPAP* continuous positive airway pressure, *COPD* chronic obstructive pulmonary disease, *BMI* body mass index, *PCOS* polycystic ovarian syndrome

^a^Female participants

**Fig. 1:**
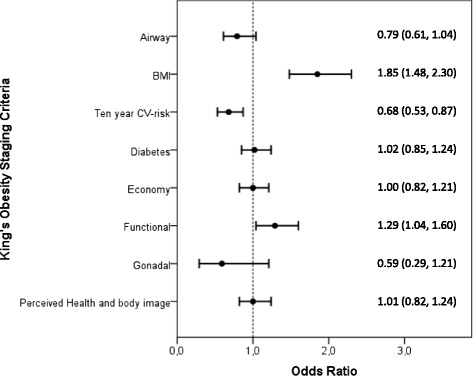
The figure shows univariable analysis for obesity treatment by each domain of King’s obesity staging criteria (KOSC)

Figure 1 shows that patients with a BMI ≥ 40 kg/m^2^ had 85% increased odds of choosing bariatric surgery, whereas physical inactivity was associated with 29% increased odds of bariatric surgery (reference lifestyle intervention). In addition, patients with ≥20% risk of CVD had 32% lower odds of bariatric surgery than those with < 20% risk of CVD. Among patients with ≥20% increased CV-risk, the 105 patients treated with bariatric surgery were on average four years younger than the 241 patients treated with intensive lifestyle intervention (age 54 [6] vs. 58 [7] years, *p* < 0.001, respectively). After adjustments for age and gender, only higher stages of BMI remained significantly associated with subsequent bariatric surgery (OR 1.81 [95% CI 1.46, 2.27]). After stratification by gender, having a BMI ≥ 40 kg/m^2^ was associated with higher odds of subsequent bariatric surgery than BMI < 40 kg/m^2^ in both women and men (OR [95% CI] 1.77 [1.37, 2.30] and 2.07 [1.38, 3.13]). Conversely, compared with being physically active for a minimum of one our each week, being physically inactive was associated with subsequent bariatric surgery in women, but not in men (1.36 [1.05, 1.78] and 1.12 [0.76, 1.65]).

Compared with patients with minor health risk in the BMI domain (Domain B, stage 1), patients within both stage 2 and stage 3 were more likely to undergo bariatric surgery (stage 2: 1.71 [1.37, 2.15] and stage 3: 3.22 [1.73, 3.14], respectively). In the diabetes domain (Domain D), conversely, patients within stage 2 (but not stage 3) were more likely to undergo bariatric surgery compared to patients without diabetes (stage 2: 1.24 [1.00, 1.54] and stage 3: 0.89 [0.57, 1.39]).

## Discussion

The main finding of this retrospective study of 2142 treatment seeking patients was that only obesity grade 3 (BMI ≥ 40 kg/m^2^) was significantly associated with increased odds of undergoing bariatric surgery as the primary treatment for morbid obesity. Conversely, increased risk of CVD was associated with lower odds of opting for surgical treatment. To the best of our knowledge this is the first study to assess, using a holistic obesity staging system, if patients opting for bariatric surgery differ from those choosing conservative treatment. Importantly, the KOSC were applied retrospectively and did not influence treatment choice. The patients and the multidisciplinary team took part in a shared decision-making process [11]. We could not confirm our hypothesis that surgical patients would have higher scores in most domains of the KOSC.

The surgical patients had a slightly lower 10-year risk of CVD (9.4% vs 10.6%) than patients who chose lifestyle intervention. This might partly be explained by the lower mean age in the surgical group, as well as by the fact that some clinicians may regard high age (e.g., > 60 years) as a relative contraindication against surgery. Our results support the notion that older patients tend to choose lifestyle intervention rather than weight loss surgery. On the other hand, compared with lifestyle intervention, undergoing bariatric surgery as treatment for morbid obesity was associated with significantly reduced mortality-risk even in patients aged 55–74 years according to a recently published study [15]. Thus, age alone should not be considered a contraindication in the preoperative risk-assessment for bariatric surgery. Importantly, although high-risk patients in general might benefit more from bariatric surgery, very high risk patients may also have an increased risk of early postoperative complications and death [16]. Accordingly, an individual’s risk of a future CV-event, as well as the postoperative risk of complications, should be systematically assessed and discussed with the patient and surgeon before a treatment decision is taken.

There is convincing evidence that bariatric surgery is associated with resolution of type 2 diabetes or improved glycemic control [17–20]. By contrast, the possible long-term beneficial effects of bariatric surgery on diabetes and its complications are less well documented. Our retrospective analysis indicates that the presence of diabetes (stage 2–3) did not significantly influence treatment choice, although patients with diabetes and an HbA1c below 9.0% (stage 2), had slightly higher odds (24%) of choosing bariatric surgery than lifestyle treatment. This might indicate that the beneficial effects of bariatric surgery have been under-communicated by our multidisciplinary teams, or that patients with advanced disease were recommended to abstain from surgery due to higher risk of postoperative complications.

A diagnosis of moderate to severe obstructive sleep apnea requiring continuous positive airway pressure (CPAP) treatment (Domain A, stage 2) was not associated with treatment choice, OR 0.79 (95% CI 0.61, 1.03). However, the prevalence of moderate to severe OSA requiring CPAP-treatment (11–14%) in the present analysis is probably underestimated as the data were retrieved at the first patient visit before the systematic work-up for possible co-morbidities (including sleep registrations). A previous clinical trial comparing gastric bypass and intensive lifestyle intervention recruiting patients from the same population showed that 35% and 25% of the patients in the surgical and lifestyle groups had moderate to severe OSA, indicating the need for CPAP-treatment [21].

Avoiding inactivity may reduce all-cause mortality, and increased levels of physical activity have been reported after bariatric surgery [22, 23]. In our analysis, patients with less than one hour physical activity per week were more likely to undergo bariatric surgery than lifestyle intervention. However, although physical inactivity was moderately associated with subsequent bariatric surgery in the univariable analysis, this association should be interpreted with caution. After adjustments for age and gender, physical inactivity was no longer significantly associated with increased odds of having bariatric surgery. Furthermore, stratification by gender showed that this association was significant in women only. Whether physical inactivity should be a criterion favoring bariatric surgery remains open for discussion.

The economy domain (Domain E) was defined according to working status as given by the public social welfare system in Norway. We were not able to distinguish between the two lower stages in Domain E, and workplace disadvantage was classified into stage 2 whereas being disabled was classified into stage 3. This classification might have overestimated the workplace disadvantage from severe obesity as other possible causes were not available. Two other studies have prospectively assessed the financial aspects of obesity before and after bariatric surgery [9, 20]. Both of these studies presented self-reported information from patients prior to surgery and 12 months after surgery. Although one of the studies reported improvements in the economy domain after surgery [20], the other did not [9].

Whether data on gonadal status may facilitate obesity treatment choice in patients with morbid obesity is not clear. In a recently published study of women with morbid obesity, androgen status normalized after gastric bypass surgery, but the hormonal changes did not reverse metabolic abnormalities [24]. The present study assessed the gonadal domain in women, with the prevalence of hyperandrogenemia and PCOS not differing significantly between treatment groups.

At present, there is no consensus on the therapeutic consequences of a psycological evaluation of patients with severe obesity. Psychological factors or motivation for obesity treatment were not measured in the present study. However, the patients in the surgery group had a longer duration of obesity and possibly a prolonged period of unsuccessful conservative obesity treatment. In accordance with the results of a previous review by Wadden et al., a large proportion (aproximately 40%) of the patients in our cohort reported a lifetime history of symptoms of either anxiety or depression, with no difference between treatment groups [25]. Although mental health and affective symptoms often improve after bariatric surgery, it is questionable whether patients with serious psychological symptoms benefit from bariatric surgery, and should rather as such abstain from surgery [26, 27]. A survey of psycological assessment of bariatric surgery applicants showed that psyhologists differed in their preoperative evaluations, with the respondents recommending either the delay or denying of surgery for between zero and 60% of the candidates. [28].

### Strengths and limitations

The major strength of this study is the large cohort of consecutively included treatment seeking Caucasian patients (97%) with morbid obesity. Limitations include the retrospective design of the study. Furthermore, the results may not be generalized to individuals of other ethnicities. Moreover, the study participants received treatment between 2005 and 2010, a period in which our multidisciplinary team tended to focus more on weight loss, whereas subsequently our focus has been more on comorbidities when discussing with patients the outcomes of treatment. Separate analysis of the missing data of domain E and F showed that the patients with missing data were comparable with patients with available data in terms of age, BMI and gender distribution (data not shown). The relatively long wait time between baseline and bariatric surgery might have favored an initial non-surgical treatment choice. On the other hand, given that the wait time for patients who chose lifestyle intervention first was even longer than for patients who chose bariatric surgery, wait time for surgery has probably not influenced the treatment choice in this study. Finally, each patient was informed about risks and benefits by a multidisciplinary team including an internist, while a surgeon was not consulted if the patient opted for non-surgical treatment. We cannot rule out that this might have favored a higher proportion of patients opting for intensive lifestyle intervention as first-line treatment.

## Conclusion

BMI was the strongest KOSC-domain associated with the choice of subsequent bariatric surgery after a shared decision making process. This study assessed the KOSC retrospectively and cannot therefore provide information on the usage of KOSC as a clinical tool designed to select patients for lifestyle intervention or bariatric surgery. Future prospective outcome studies are necessary to assess the applicability of the KOSC in terms of supporting the most appropriate treatment choice as a part of the shared decision making process.

## Abbreviations

BMI: Body mass index
CV: Cardiovascular
DM: Diabetes mellitus
KOSC: Kings obesity staging criteria
MeTs: Metabolic syndrome
PCOS: Polycystic ovarial syndrome
